# ATG12–ATG5-TECPR1: an alternative E3-like complex utilized during the cellular response to lysosomal membrane damage

**DOI:** 10.1080/15548627.2023.2267414

**Published:** 2024-01-25

**Authors:** Dale P. Corkery, Yao-Wen Wu

**Affiliations:** a SciLifeLab and Department of Chemistry, Umeå University, Umeå, Sweden; b Umeå Centre for Microbial Research, Umeå University, Umeå, Sweden

**Keywords:** ESCRT, lysophagy, lysosome, membrane repair, TECPR1

## Abstract

ATG16L1 is an essential component of the Atg8-family protein conjugation machinery, providing membrane targeting for the ATG12–ATG5 conjugate. Recently, we identified an alternative E3-like complex that functions independently of ATG16L1. This complex utilizes the autophagosome-lysosome tethering factor TECPR1 for membrane targeting. TECPR1 is recruited to damaged lysosomal membranes via a direct interaction with sphingomyelin. At the damaged membrane, TECPR1 assembles into an E3-like complex with ATG12–ATG5 to regulate unconventional LC3 lipidation and promote efficient lysosomal repair.

Lipidation and membrane anchoring of the Atg8 family of ubiquitin-like proteins represents an essential event in both canonical and noncanonical autophagic processes. This process involves the conjugation of Atg8-family proteins to phosphatidylethanolamine/PE or phosphatidylserine/PS, orchestrated by a ubiquitin-like conjugation system composed of ATG7 (E1), ATG3 (E2) and the ATG12–ATG5-ATG16L1 complex (E3). Within the E3-like complex, the ATG12–ATG5 conjugate possesses the E3 ligase-like activity, while ATG16L1 is largely responsible for target membrane recognition. Depletion of ATG16L1 prevents the recruitment of ATG12–ATG5 to membranes, impeding subsequent Atg8-family protein conjugation. Thus, ATG16L1 is deemed indispensable for Atg8-family protein lipidation.

Recently, we identified an ATG16L1-independent E3-like complex which utilizes TECPR1 (tectonin beta-propeller repeat containing 1) for membrane recognition [1]. TECPR1, a resident lysosomal protein, promotes autophagosome-lysosome fusion via interaction with the ATG12–ATG5 conjugate on autophagosomal membranes. Our study began with the observation that TECPR1 is recruited to lysosomes in response to membrane damage induced by L-Leucyl-L-Leucine methyl ester (LLOMe) or glycyl-L-phenylalanine 2-naphthylamide/GPN. To characterize the region of TECPR1 responsible for damage-induced lysosomal recruitment, we generated a series of TECPR1 mutants and assed their ability to translocate to lysosomes upon LLOMe treatment. Using this approach, we identified the N-terminal dysferlin domain of TECPR1 as essential for its lysosomal recruitment. Furthermore, we showed that interaction between the N-terminal dysferlin domain of TECPR1 and sphingomyelin on the damaged membrane is directly responsible for TECPR1’s recruitment to damaged lysosomes.

How a cell responds to lysosomal damage depends in large part on the extent of the damage itself. Small disruptions are detected and repaired by the endosomal sorting complex required for transport (ESCRT) machinery, while more severe membrane damage is resolved via lysophagy. Sphingomyelin scrambling at damaged membranes has been reported to precede membrane rupture, consistent with our observation that TECPR1 is recruited to damaged lysosomes prior to LGALS3 (galectin 3), a protein that binds to intralumenal glycans exposed by membrane rupture. Furthermore, our findings reveal that TECPR1 is co-recruited to damaged lysosomes with the ESCRT machinery indicating a potential role in membrane repair rather than membrane removal.

TECPR1 and ATG16L1 form mutually exclusive complexes with the ATG12–ATG5 conjugate, which prompted us to look at how TECPR1 recruitment affects Atg8-family protein lipidation at the damaged membrane. To our surprise, we observed LC3 lipidation occurring in multiple *ATG16L1* KO cell lines following LLOMe treatment, despite ATG16L1 being long considered an essential component of the Atg8-family protein conjugation system. Immunofluorescence microscopy combined with LysoIP confirmed that, in the absence of ATG16L1, LC3 was being conjugated to damaged lysosomal membranes. This observation suggested that TECPR1 might be forming an alternative E3-like complex with the ATG12–ATG5 conjugate. Double knockout of *ATG16L1* and *TECPR1* fully abolished LLOMe-induced LC3 lipidation, conclusively demonstrating that ATG12–ATG5-TECPR1 acts as an alternative E3-like complex to regulate Atg8-family protein lipidation in response to lysosomal membrane damage.

The conventional ATG12–ATG5-ATG16L1 E3-like complex is generally considered to be an essential component of the lysophagy pathway, responsible for the removal of excessively damaged membranes. The sphingomyelin-dependent recruitment of TECPR1 places the alternative E3-like complex upstream of membrane rupture, suggesting a role in membrane repair. To gain insight into the function of TECPR1 at the damaged membranes, we performed a lysosome repair assay. In this assay, lysosomes were acutely damaged with an LLOMe pulse, followed by washout and recovery. The restoration of lysosome function after damage was assessed via LysoTracker staining. The results showed that individual knockout of either *ATG16L1* or *TECPR1* had a minimal effect on lysosomal recovery, whereas the double knockout significantly impaired the membrane repair process. This suggests that both E3-like complexes play a functionally redundant role in lysosomal membrane repair.

Collectively, our findings identify ATG12–ATG5-TECPR1 as an alternative E3-like complex which functions independently of ATG16L1 to regulate unconventional LC3 lipidation at damaged lysosomal membranes and promote efficient membrane repair (Figure 1). Ongoing work aims to identify the mechanism through which TECPR1 contributes to the repair process.

**Figure 1.**
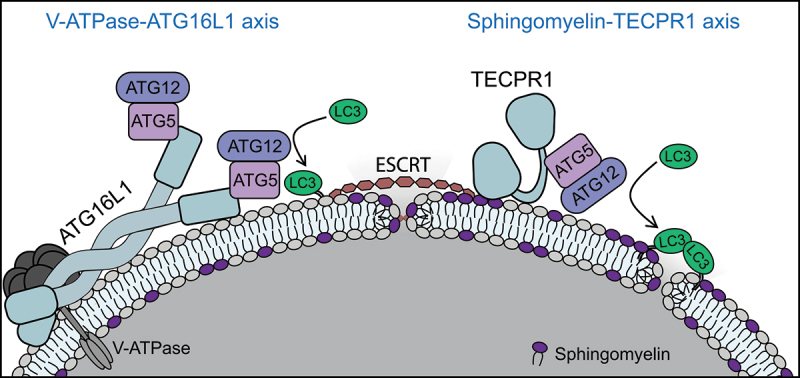
Model of Atg8-family protein conjugation to damaged lysosomal membranes by two distinct E3-like complexes.

## References

[1] Corkery DP, Castro-Gonzalez S, Knyazeva A, et al. An ATG12-ATG5-TECPR1 E3-like complex regulates unconventional LC3 lipidation at damaged lysosomes. EMBO Rep. 2023;24:e56841. doi: 10.15252/embr.202356841 37381828 PMC10481663

